# Development of a Contactless Air Conveyor System for Transporting and Positioning Planar Objects

**DOI:** 10.3390/mi9100487

**Published:** 2018-09-24

**Authors:** Xirui Chen, Wei Zhong, Chong Li, Jiwen Fang, Fanghua Liu

**Affiliations:** 1 School of Mechanical Engineering, Jiangsu University of Science and Technology, Zhenjiang 212003, China; cxrfight1227@gmail.com (X.C.); lichong@just.edu.cn (C.L.); fjw617@just.edu.cn (J.F.); cylfhua@just.edu.cn (F.L.); 2 Jiangsu Provincial Key Laboratory of Advanced Manufacture and Process for Marine Mechanical Equipment, Jiangsu University of Science and Technology, Zhenjiang 212003, China

**Keywords:** air conveyor, viscous force, contactless transport, pressure distribution, position control

## Abstract

In this study, we developed a completely contactless air conveyor system for transporting and positioning planar objects. The air conveyor forms a thin film underneath the object for support and simultaneously generates a controlled airflow that results in viscous traction. It is potentially applicable in the manufacturing process for semiconductor wafer or flat foodstuffs, where mechanical contact is expected to be avoided during transportation of the products to minimize contamination. The air conveyor employs duplicated arrays of actuating cells that are square pockets with a surrounding dam. A simple model is proposed to characterize the viscous force. The theoretical analysis reveals that the total force is the composition of an actuating force generated in the pocket areas and the side areas and a drag force generated in the dam areas. Experimental investigations are conducted on the basic characteristics of the film pressure distribution and the viscous force. The results show that the air film pressure is symmetrically distributed in the width direction but nonsymmetrically distributed in the length direction. The viscous force increases if the suction flow rate is enlarged or the gap thickness is narrowed. Comparison of the experimental results and the calculated results indicates that the model can provide an accurate prediction. A proportional–integral–derivative (PID) controller is applied for 1D-position control and position tracking. The actuating direction is selected using fast switching valves and the amplitude of the actuating force is adjusted using a control valve to vary the suction flow rate. The simulated and the experimental results verify the feasibility of the air conveyor system and the control method.

## 1. Introduction

Many industries require contactless transport of delicate or clean products such as silicon wafers, flat foodstuffs, and freshly painted objects. Transport by means of pneumatics technology is widely used in practical applications since it is clean, free from magnetism, and generates little heat [1,2,3]. Noncontact vacuum grippers [4,5,6,7,8] and air-cushion conveyors [9,10,11,12,13] are the most commonly used methods to avoid mechanical contact. The vacuum grippers generate an upward lifting force and thereby can pick-and-place a planar object. However, an object handled in this way is very likely to drop when tilted and thus locating pins or rubber friction pads are generally equipped on the grippers. The air-cushion conveyor uses arrays of bearing elements which supply pressurized air to form an air film under the object. The two methods are similar in that the object is levitated at an equilibrium position where its weight is balanced by the lifting force. Unfortunately, both methods cannot completely avoid backside contact when moving the object in the horizontal direction.

The conventional air-cushion method forms an air film to support the object but cannot provide a horizontal actuating force because the supplied airflow is perpendicular to the surface. Both the actuating function and the supporting function can be realized if a tilted airflow relative to the surface is in use. Paivanas et al. [14] described a system for wafer handling and transport applications using inclined air jets, and Biegelsen et al. [15] developed a paper mover using directed air jets. However, in these cases, the apertures are fixed and the object can only be moved in a predetermined direction that cannot be easily realized if the object needs to change direction. Therefore, researchers have found approaches to improve moving mechanisms. Konishi and Fujita [16] designed a fluidic microactuator which involves two on-off nozzles to transport tiny objects by changing the airflow direction. Zeggari et al. [17] presented the design and testing of a pneumatic conveyor which can levitate and move flat objects to a desired position using tilted air-flow. Fukuta et al. [18] reported the design, fabrication, and control of arrayed microelectromechanical systems (MEMS)-based actuators for distributed micromanipulation by generation and control of the air-flow force field. D. El Baz et al. [19] introduced a smart surface for conveying and positioning microparts; they established a mathematical model for discrete state acquisition and proposed several distributed synchronous and asynchronous discrete state acquisition algorithms. 

In addition to tilted air jets, some researchers used the aerodynamic-traction principle to move objects. Airflow across the surface of an object induces shear stress at the boundary and this might be used for actuation. Chen et al. [20] used viscous shear by air flow around a rotor shaft to drive a spindle, and Delhaes [21] presented a high-speed spindle which is both driven and supported by a viscous turbine. In these cases, the spindle is driven by exerting viscous force on the smooth surface, and similarly, the same principle can be applied to linear actuation. Moon and Luntz [22] accomplished this by generating the manipulation flow field on the top surface of an object while a standard air table generates an air cushion for support. Delettre [23] developed a 120 mm × 120 mm positioning system which uses individually controlled vertical jets for propulsion. Laurent et al. [24] utilized this system to perform experiments with several objects, but unfortunately they did not present a model for viscous force as a function of gap thickness. Ku et al. [25] developed a surface device using a 4 mm × 4 mm array of 100 capillary glass tubes which are individually connected to valves to generate a controllable pressure field. 

The Mechatronics System Design Group at the Technical University of Delft (TU Delft) has conducted long-term and meticulous research on product transport by means of using arrays of actuator cells to produce horizontal air flow underneath the object. The idea was originally raised by van Ostaijen in the patent published in 2008 [26]. The patent introduces a kind of air actuator which carries and transports a planar product by forming a thin layer of compressed air underneath the substrate to float and move the object in a desired direction. Afterwards the research group continuously advanced this research. In 2008, van Rij et al. [27] explained the operating principles of the new contactless transport system and its detailed mathematical modeling process. The computation results indicated that the generated acceleration approximates 2.5 m/s^2^ for a glass substrate with a surface area of 2 m^2^ and a thickness of 0.7 mm. In 2009, van Rij et al. [28] optimized the air actuator by means of grooves connecting the inlet points and outlet points; the simulation results by a finite element method (FEM) model indicated that more traction force can be generated after optimization. Later, van Ostayen et al. [29] and Jasper Wesselingh et al. [30] comprehensively studied and detailed the theory, modeling, design, implementation, testing, and evaluation of the developed air actuator. In 2017, Krijnen et al. [31] developed a novel contactless positioning system for silicon wafers with the application of fractional-order proportional–integral–derivative (PID). Their research on the topic of product transport continues currently and more results will be reported in future. 

Objectively, the systematic work by the research group of TU Delft has greatly enriched the implementation of contactless transport using a pneumatic approach. The present work is greatly inspired by the previous research of TU Delft and actually follows some of the ideas in mathematical modeling and apparatus design. In this paper, we have designed a completely contactless air conveyor system for object transportation and positioning in a similar way as previously published [30]. First, a simplified theoretical model is deduced and used for calculating the viscous force. Next, the film pressure distribution and viscous force are experimentally measured. Then, a detailed analysis of the film pressure distribution and viscous force is provided. Finally, theoretical and experimental results with a PID controller—used for dimension position control and position tracking—are presented. 

## 2. Mechanism of the Air Conveyor 

**Figure 1:**
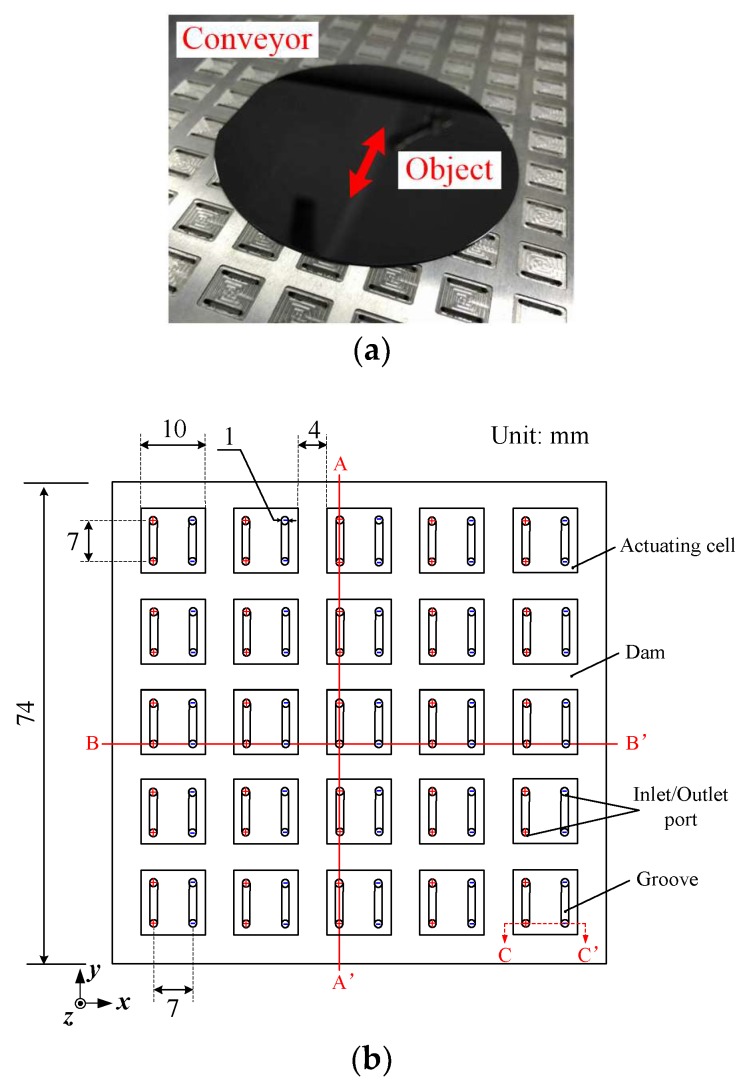

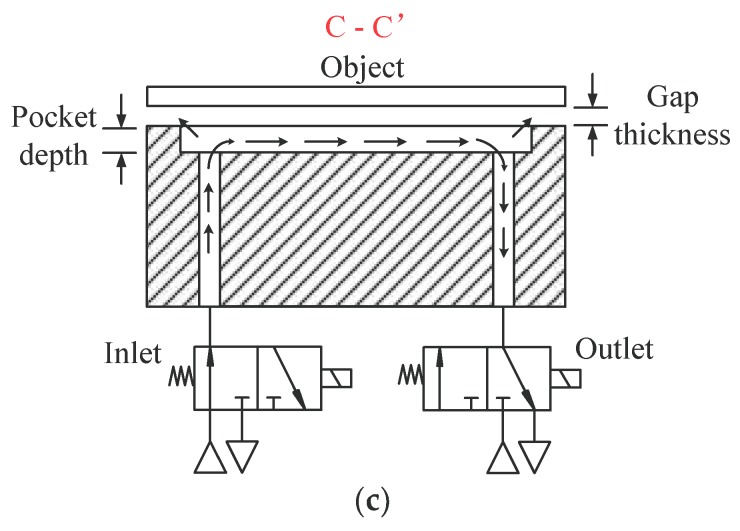
Air conveyor. (**a**) Photograph of the air conveyor. (**b**) A representative unit consisting of 25 actuating cells. (**c**) Cutaway view that shows the working principle.

Figure 1a shows a photograph of the air conveyor. A flat object is levitated by the formed air film, and can be moved and positioned using controlled airflow in the gap. The air conveyor employs duplicated arrays of actuating cells. To facilitate analysis and experiments, as shown in Figure 1b, a representative unit is focused. The unit is a 74 mm square consisting of 25 actuating cells. Each cell is actually a 10 mm square pocket (depth: 150 μm) surrounded by a dam with a width of 4 mm. In the pocket, two pairs of inlet/outlet ports (φ = 1 in diameter) are opened. The two inlet ports are connected with a groove, which has a dimension of 1 mm width and 1 mm depth, and similarly, the two outlet ports are also connected with a groove. We consider that these grooves can stabilize the flow in the pocket. In this study, we aim to achieve one dimensional transport and positioning. Accordingly, all the inlet/outlet ports located in the same column are connected with an internal perforation which is then connected to an independent fast switching valve (3/2 normally opened valve). By this means, each column can be selected for positive pressure or negative pressure by switching the valves as required. 

The working principle is illustrated in Figure 1c. The inlet ports are connected to positive pressure while the outlet ports are connected to negative pressure. Therefore, a horizontal airflow is formed in the pocket due to the pressure difference that accordingly generates a viscous force, namely, the shear stress at the boundary. Meanwhile, the entering air forms a supporting film underneath the object. Because of the supporting function and the actuating function, the object can be transported without mechanical contact. 

The fabrication process of the device is described as follows. The material used was 7050 aviation aluminum-alloy. First, a cuboid-shaped plate, with dimensions of 204 mm × 228 mm × 20.15 mm, was cut down from the raw material. The upper and the lateral surfaces of the plate were roughly polished to a roughness of *Ra =* 6.3 μm. Then, thirty-two internal perforations, with a diameter of 4 mm, were drilled at intervals of 7 mm into the lateral side of the plate. Next, 16 × 8 square pockets distributed in the arrays were fabricated on the upper surface of the plate by means of a milling process, with a temporary depth of 300 μm. In each pocket, two grooves were milled according to the locations and sizes shown in Figure 1, and two pairs of inlet/outlet ports at two ends of the grooves were drilled until they were in connection with the internal perforation. Finally, the plate underwent a finish machining process to ensure a roughness of *Ra =* 0.8 μm on the upper surface and a final depth of the pockets of 150 μm.

## 3. Theoretical Modeling

It is easy to understand that establishing a theoretical model based on Reynolds equation coupling and incompressible Navier–Stokes equation could help analyze and understand certain basic characteristics, such as film pressure distribution and viscous force. However, solving the model with a finite difference method requires a large amount of calculating time and therefore such a model can never be used for motion control of the object in a real scenario. Therefore, there exists a crucial need to develop simple mathematical models with good real-time performance. In a past paper [30], Jasper Wesselingh proposed correlating equations based on the fundamental flow regime to predict the viscous force. This method greatly simplified the flow analysis on duplicated arrays of the actuating cells and proved to be effective. Therefore, we followed their treatments for region segmentation and the method of modeling in this work. 

As shown in Figure 1b, a 3D-Cartesian coordinate is established at the bottom left point. The length, width, and thickness directions are denoted as *x*, *y*, and *z* coordinates, respectively. In this study, the actuating force is only considered in the *x*-direction. Owing to the complexity of flow in the gap, the following assumptions are made to simplify the theoretical modeling.

1. Airflow in the gap is laminar, dominated by viscous effects.
2. Pressure distribution in the *z*-direction is negligible.

Consequently, the Navier–Stokes equation in the *x*-direction is simplified as the following.

(1) $$-\frac{\partial p}{\partial x}+\mu \frac{\partial^{2}u}{\partial z^{2}}=0$$

where *p* is the film pressure, *μ* is the air viscosity, and *u* is the flow velocity. 

With the boundary condition that *u =* 0 (*z =* 0) and *u = V*_0_ (*z = h*), integrating Equation (1) with respect to *z* yields:

(2) $$u=\frac{p_{2}-p_{1}}{2\mu l}\left(z^{2}-hz\right)+\frac{V_{0}}{h}z\;$$

where *p*_1_ is the pressure of the inlet ports, *p*_2_ is the pressure of the outlet ports, and *l* is the length of the pocket. 

The shear stress *τ* is linearly proportional to the velocity gradient as below.

(3) $$\tau =-\mu \frac{\partial u}{\partial z}$$

**Figure 2:**
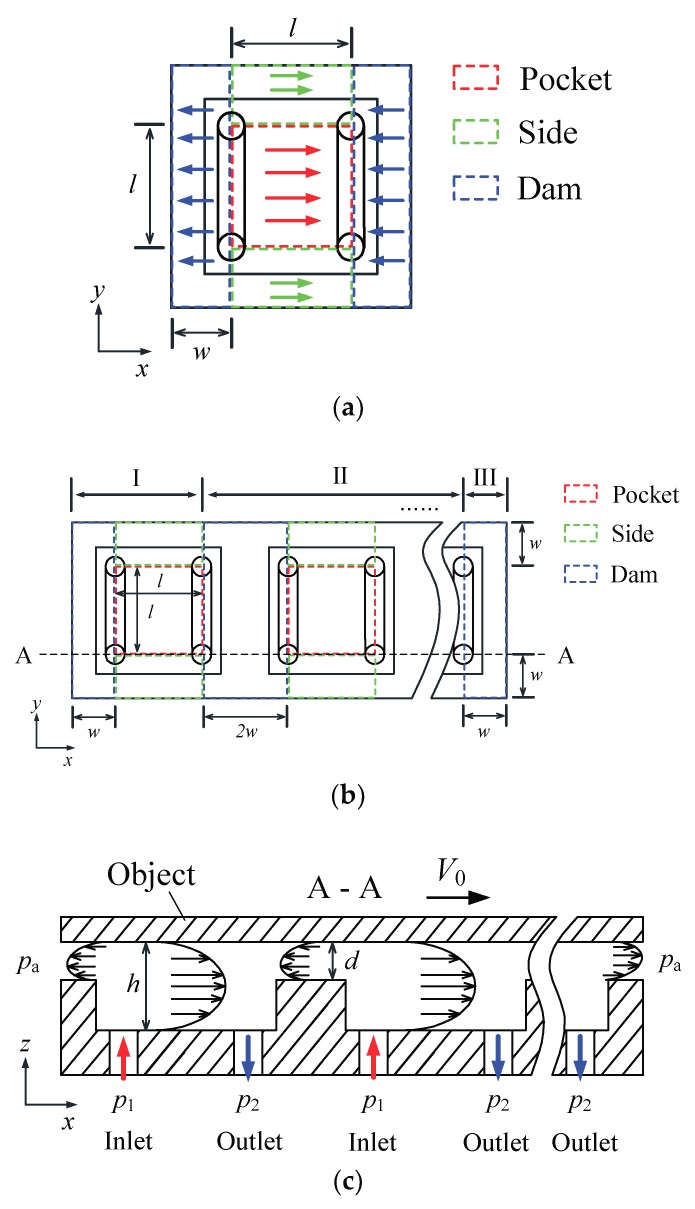
Typical cells and modeling of the airflow in the gap. (**a**) Area segmentation of a typical cell. (**b**) Area segmentation of a representative row. (**c**) Velocity profile in the gap.

Figure 2a shows a typical actuating cell consisting of three areas. They are the pocket area, dam area, and side area. The airflow in the pocket area and the side area is assumed along the *x*-direction, and the airflow in the dam area is the opposite. The film thickness for the dam area and the pocket area is denoted as *d* and *h*, respectively. Here, *d* is the distance from the lower surface of the floating object to the upper surface of the air conveyor, while *h* is the distance from the lower surface of the floating object to the surface of the pocket (refer to Figure 2c). 

With the boundary condition that *z = h*, submitting Equation (2) into Equation (3) obtains the shear stress *τ*_p_ in the pocket area.

(4) $$\tau_{p}=\frac{p_{1}-p_{2}}{l}\frac{h}{2}-\frac{\mu }{h}V_{0}\;$$

Similarly, the shear stress *τ*_d_ in the dam area is obtained as below.

(5) $$\tau_{d}=-\left(\frac{p_{1}-p_{2}}{2w}\frac{d}{2}+\frac{\mu }{d}V_{0}\right)$$

The shear stress, *τ*_s_, in the side area is thus expressed as:

(6) $$\tau_{s}=\frac{p_{1}-p_{2}}{l}\frac{d}{2}-\frac{\mu }{d}V_{0}\;$$

For multiple cells in a representative row, as shown in Figure 2b, the row is divided into three zones, designated as I, II, and III, respectively. Zone I is the leftmost cell that consists of a pocket area, a dam area, and two side areas. Zone II contains middle reduplicate cells which have similarity to that of zone I. Zone III is the rightmost dam area. 

The total viscous force is the composition of the force in the pocket, dam, and side areas (*F*_p_, *F*_d_, and *F*_s_). For zone I, the viscous force can be calculated by Equations (7)–(9), respectively.

(7) $$F_{p}=\frac{p_{1}-p_{2}}{2}hl-\frac{\mu }{h}V_{0}l^{2}\;$$

(8) $$F_{d}=-\left(\frac{p_{1}-p_{a}}{w}\frac{d}{2}+\frac{\mu }{d}V_{0}\right)\times \left[w\times \left(l+2w\right)\right]$$

(9) $$F_{s}=\left(\frac{p_{1}-p_{2}}{l}\frac{d}{2}-\frac{\mu }{d}V_{0}\right)\times 2wl\;$$

For zone II, the viscous force (*F*_p_, *F*_d_, and *F*_s_) is calculated by Equations (10)–(12), respectively.

(10) $$F_{p}=\frac{p_{1}-p_{2}}{2}hl-\frac{\mu }{h}V_{0}l^{2}\;$$

(11) $$F_{d}=-\left(\frac{p_{1}-p_{2}}{2w}\frac{d}{2}+\frac{\mu }{d}V_{0}\right)\times \left[2w\times \left(l+2w\right)\right]\;$$

(12) $$F_{s}=\left(\frac{p_{1}-p_{2}}{l}\frac{d}{2}-\frac{\mu }{d}V_{0}\right)\times 2wl\;$$

For zone III, the viscous force is calculated only in the dam area by Equation (13).

(13) $$F_{d}=-\left(\frac{p_{a}-p_{2}}{w}\frac{d}{2}+\frac{\mu }{d}V_{0}\right)\times \left[w\times \left(l+2w\right)\right]$$

As an example for the case of a covered region including *M* × *N* (Width × Length) cells in an array, the total force *F* is calculated as below,

(14) $$F=M\times F_{I}+M\times （N-1）\times F_{\mathrm{II}}+M\times F_{\mathrm{III}}\;$$

where *F*_I_, *F*_II_, and *F*_III_ denotes the viscous force of zones I, II, and III, respectively.

Figure 2c shows the velocity profile in the gap. The film pressure at the edge remains at atmospheric pressure (*p*_a_). The object is moved by an actuating force formed in the pocket area and the side area, but is also simultaneously subjected to a drag force formed in the dam area. This does not include the rightmost dam area where air exhausts to the atmosphere in the *x*-direction and exerts positive effects on the movement.

Integrating Equation (2) with respect to cross-sectional area obtains the volumetric flow rate *Q*_p_ in the pocket area.

(15) $$Q_{p}=\frac{p_{1}-p_{2}}{12\mu }h^{3}+\frac{hl}{2}V_{0}$$

The theoretical deduction of the equations above is greatly inspired by works in the literature [27,28,29,30]. When applying the equations to calculate the viscous force, there is a problem that the effective flow rate through the gap is necessary for calculating the pressure difference *p*_1_-*p*_2_. We thus made an extension to the modeling to accommodate for this in the present study. A fitting equation (Equation 16) is proposed to approximate the effective flow rate through the gap including the effect from the inlet/outlet flow and the gap thickness. The fitting coefficients are determined experimentally. This treatment improves the modeling and is especially appropriate for the present study since the position control of the object is implemented by varying the suction flow rate (detailed in Section 5.2).

(16) $$Q_{p}=\left(\alpha Q_{1}+\beta Q_{2}\right){\left(\frac{d_{0}}{d_{0}+\delta d}\right)}^{n}\;$$

where *Q*_1_ is the inlet flow rate, *Q*_2_ is the suction flow rate, *α*, *β*, and *n* are undetermined parameters, *d*_0_ is the initial thickness, and *δd* is the changed thickness.

It should also be noted that in the actual case variation of the suction flow rate would affect the floating height of the object. However, the accompanying effect was not included in the theoretical model but its inclusion would be expected in future work.

## 4. Basic Characteristics 

### 4.1. Pressure Distribution

**Figure 3:**
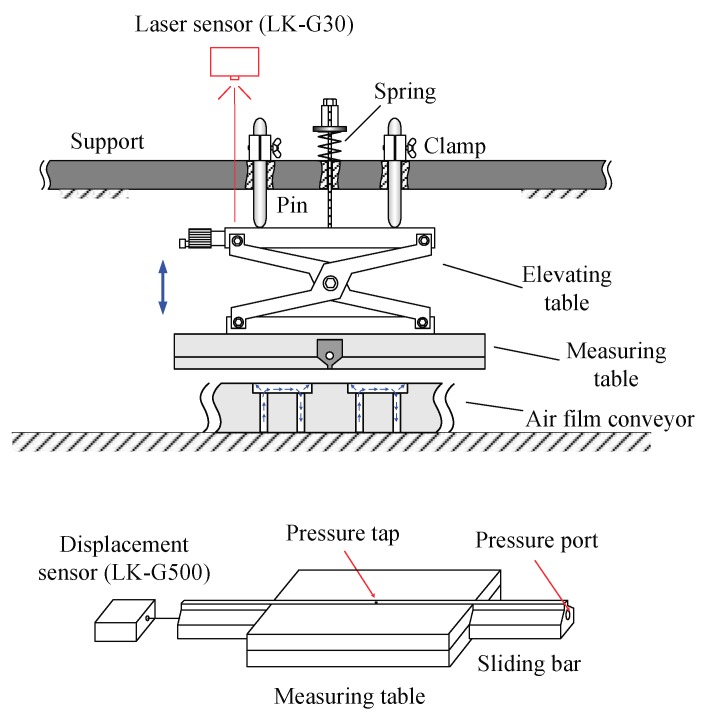
Schematic of the apparatus for pressure distribution measurement.

Since pressure distribution is very sensitive to inclination of the gap, the measuring table must be strictly parallel with the test device and the gap thickness should be finely adjusted. An experimental apparatus is proposed to accomplish this, as schematically shown in Figure 3. A measuring table with a sliding bar which has a small tap hole and an internal connecting perforation was used to measure the film pressure. First, the measuring table was installed at the bottom of an elevating table. Then, the measuring table was fixed by the following steps: (1) The measuring table was placed inversely on the conveyor surface and then set down three pins to make sure that their tips touched the top of the elevating table. (2) The pin clamps we turned until the pins were held tightly, and then we used a spring to fix the elevating table to the support. The vertical position of the measuring table is adjustable and can be read through a laser displacement sensor (LK-G30, Keyence Co., Ltd., Osaka, Japan, resolution: 0.1 μm). Another displacement sensor was used to measure the position of the sliding bar. A pressure sensor (KL17, Nagano Keiki Co., Ltd., Tokyo, Japan) with a range of 0–2 kPa was connected to the pressure tap through the internal perforation. In this way, the pressure distribution can be measured by recording the film pressure and position while slowly moving the sliding bar. 

**Figure 4:**
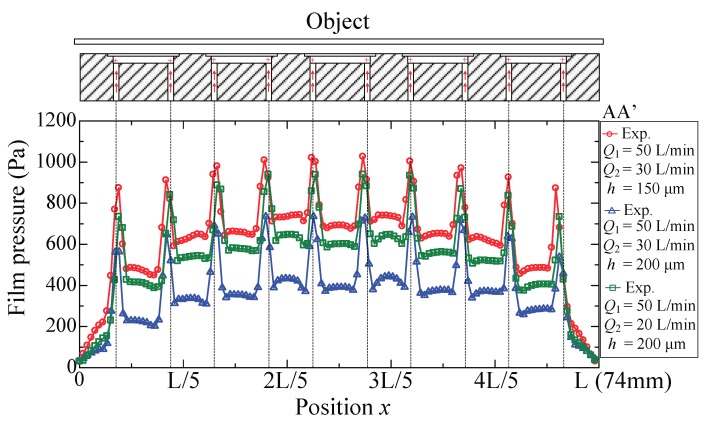
Film pressure distribution.

Experiments were performed with supply flow rate *Q*_1_ = 50 L/min (ANR), suction flow rate *Q*_2_ = 20 and 30 L/min (ANR), and gap thickness *h =* 150 and 200 μm, respectively. Figure 4 shows the film pressure distribution on line AA’ (cf. Figure 1b), which is perpendicular to the direction of airflow in the pockets. In each pocket, there are two high pressure peaks concentrated around the center of the inlet ports. The film pressure is distributed symmetrically with respect to the centerline, and a uniform distribution is recognizable in the grooves. The amplitude of the pressure is influenced by the suction flow rate and the gap thickness. Contrast experiments were performed to clarify their effects. When the gap is narrowed, the pressure distribution shifts upward but maintains a nearly unchanged shape. This is because the viscous impedance of the gap is determined by the clearance. The narrower the clearance, the larger the viscous impedance; hence, the entire film pressure increases as the gap impedance is enlarged. If the suction flow rate, *Q*_2_, is enlarged, the amount of air that escapes into the dam area would reduce and thus lead to decreased pressure distribution. The symmetrical pressure distribution indicates that the viscous force generated in the width direction is considerably small and therefore can be neglected.

**Figure 5:**
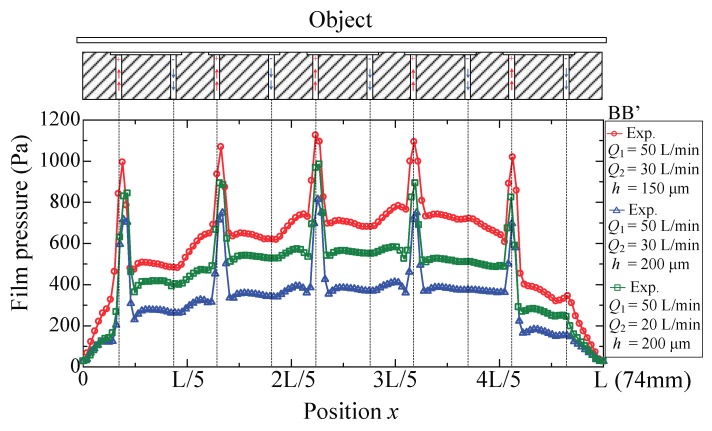
Film pressure distribution.

Figure 5 shows the film pressure distribution on line BB’ (cf. Figure 1b), which is distributed along the direction of the airflow in the pockets. In each pocket, there is only one high pressure peak at the center of the inlet ports, and the pressure distribution is asymmetrical with respect to the centerline. The pressure around the outlet port is apparently lower compared with that of the inlet port; however, it is still higher than the atmospheric pressure. As the gap thickness or the suction flow rate changes, the pressure distribution shifts upward or downward but also maintains a nearly unchanged shape. As is well-known, the pressure distribution shape reflects the flow behavior. In the pockets, the decreasing pressure indicates that air flows from the inlet ports to the outlet ports. The generated airflow therefore produces a force that actuates the object. In the dam area, the pressure gradient shows an opposite trend because of the airflow from the neighboring cells. This confirms that the airflow across the dam area is actually counteractive to the movements of the object.

### 4.2. Viscous Force

**Figure 6:**
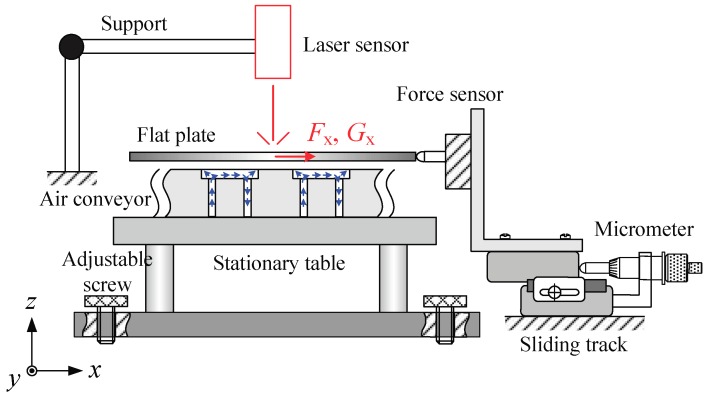
Schematic of the apparatus for viscous force measurement.

Measuring the viscous force is important since it is an effective way to evaluate the accuracy of the mathematical model. The research group of TU Delft [30] reported a well-designed experimental apparatus which ensures adequate measuring accuracy of viscous force in the order of 10^−3^ N. In this work, the experimental apparatus is designed in a similar way as schematically shown in Figure 6. A flat plate is supported upon the conveyor surface by a pressurized air film. A force sensor (LVS-5GA, Kyowa Electronic Instruments, Tokyo, Japan) is installed on a sliding track, and its mounting head contacts with the lateral side of the plate to measure viscous force. The force sensor was calibrated and a preamplifier was used. The position of the sliding track can be finely adjusted using the micrometers. The air conveyor unit is placed on a stationary table, which is mounted on a slight tilt by adjusting three adjustable screws. Thus, the mounting head of the force sensor is in contact with the plate, and viscous force measurement can be obtained by subtracting the component of gravity of the plate. Moreover, the film thickness can be varied by slowly changing the mass of the floating object and detected using a laser displacement sensor (LK-G30, Keyence Co., Ltd., Osaka, Japan, resolution: 0.1 μm). 

**Figure 7:**
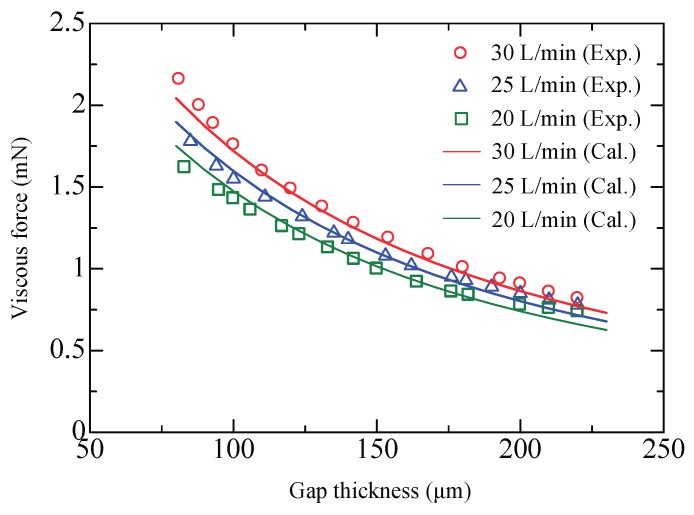
Viscous force versus gap thickness.

During the experiments, all the inlet ports in the same column were connected with positive pressure and the outlet ports with negative pressure. The supply flow rate *Q*_1_ was fixed at 40 L/min while the suction flow rate *Q*_2_ was set to 20, 25, and 30 L/min. Figure 7 plots the measured viscous force *F* versus the gap thickness *h*. Clearly, the *F*–*h* curve moves upward as the suction flow rate increases. With a constant suction flow rate, the viscous force decreases with gap thickness. Airflow in the pockets is accelerated by enhancing the suction flow or reducing the gap thickness and thus a larger viscous force can thus be generated. The calculated results were obtained using Equations (14)–(16). First, the effective flow rate through the gap in the pocket areas is estimated by Equation (16). Then, with Equation (15), the pressure difference *p*_1_-*p*_2_ is obtained. Equation (14) is the integration of Equations (7)–(13) representing the sum of generated viscous force. Observations in Equations (7)–(13) show that if *p*_1_-*p*_2_ is known, the viscous force with different flow rate can be calculated. The fitting coefficients in Equation (16) are experimentally determined as *α* = *β* = 0.12 and *n* = 0.03. An error less than 5% is estimated by the root-sum-square method indicating that the calculated results afford sufficient accuracy.

**Figure 8:**
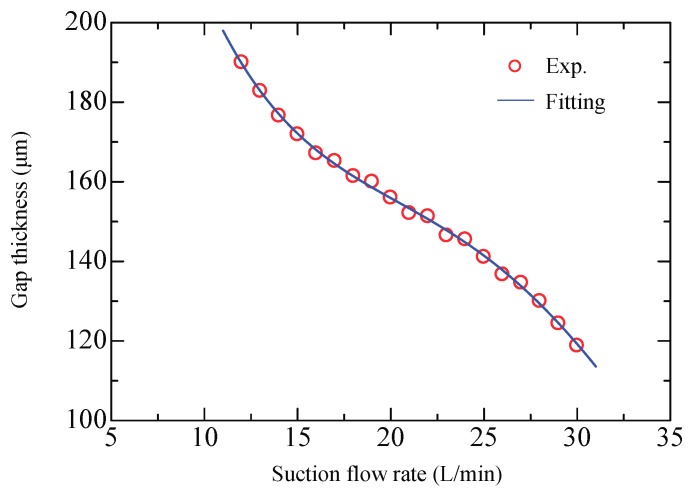
Gap thickness versus suction flow rate.

The floating height of the plate is actually influenced by the suction flow rate. Figure 8 shows the relation for a plate of weight 89.5 g. From the results of the pressure distribution it is clear that film pressure changes with suction flow rate. Obviously, the plate would float at a lower height with greater suction flow rate. So if we use the theoretical model to correlate the viscous force with the suction flow rate, we should take into account the resulting variation in gap thickness. Accordingly, an approximate curve was fitted to the data points using a 4th-order polynomial regression (*R*^2^ > 0.99) to represent the relation of *h*-*Q*_2_. 

**Figure 9:**
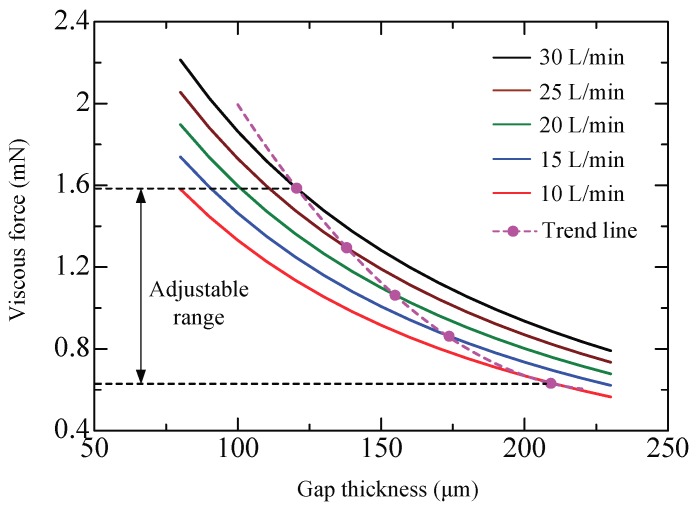
Calculated viscous force versus gap thickness.

Figure 9 shows the calculated viscous force versus gap thickness with different suction flow rate *Q*_2_. For the motion control of the object, the viscous force is expected to be variable and this can be achieved by connecting a control valve to the outlet port to vary the suction flow rate. Increasing the suction flow rate and decreasing the gap thickness both exert a positive effect on the viscous force. Therefore, when *Q*_2_ increases, the viscous force actually exhibits a more significant upward trend due to the accompanied decrease in gap thickness. An adjustable range of the viscous force can be discerned according to the upper and lower limits of the suction flow rate. Furthermore, since the trend curve heavily relies on the relation of *h*-*Q*_2_, it would move left or right depending on whether the weight of the object changes.

**Figure 10:**
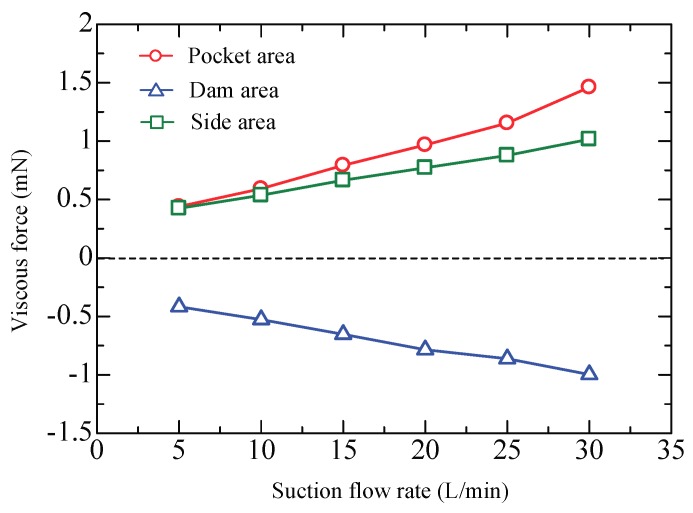
Viscous force contributed by different areas.

The total viscous force is the composition of the actions in the pocket areas, dam areas, and side areas. Figure 10 plots the calculated viscous force versus the suction flow rate to demonstrate the contributions of different areas. The positive value means that the airflow in the pocket areas and the side areas produces an actuating force, while the negative value for the dam areas represents a drag force. Clearly, both the actuating force and the drag force show an increasing trend with suction flow rate, and, the greatest contribution is from the pocket areas. Careful observation shows that the force contributed by the side areas is almost in equilibrium with that contributed by the dam areas. This means that the theoretical model can be further simplified, only considering the effects from the pocket areas.

## 5. Position Control

### 5.1. Experimental Setup

**Figure 11:**
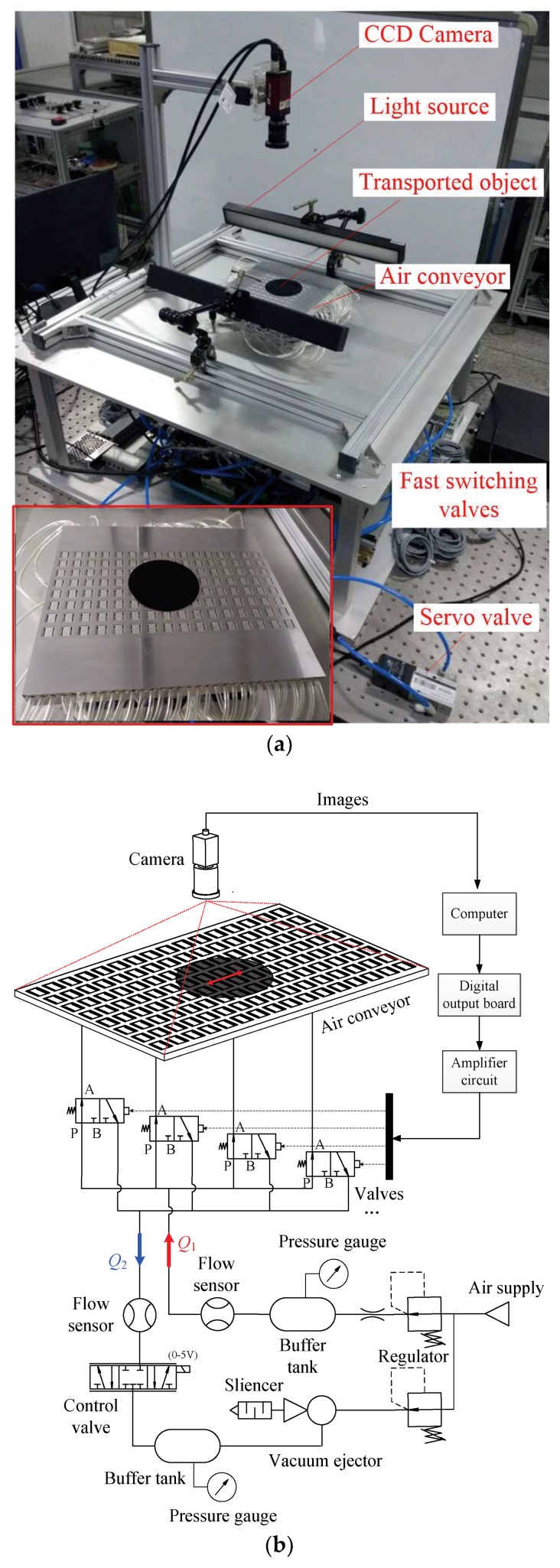
(**a**) Photograph of the experimental setup. (**b**) Air supply circuit.

Figure 11a shows the photograph of the experimental apparatus for position control of a planar object. The air conveyor is a 228 mm × 204 mm rectangle consisting of 128 actuating cells. The upper surface was carefully leveled by turning the supporting screws. A charge-coupled device (CCD) Camera (Manta G-031B, Allied Vision, Stadtroda, Germany) was placed above the conveyor to obtain the position of the object. Light sources were installed to improve the accuracy of the vision detection. Figure 11b shows the air supply circuit, which includes a pressurized air branch and a vacuum branch. The vacuum branch was created using a vacuum generator (vacuum ejector or vacuum pump). Two buffer tanks were placed to stabilize the supplied positive pressure and the negative pressure, respectively. For 1D-position control, the inlet/outlet ports that share a column are interconnected and can be selected for the air circuit or the vacuum circuit using a 3/2 fast switching valve (MHA3-M1H-3/2G-3-K, Festo Co., Ltd., Esslingen, Germany); a total of 32 valves are in use. Ports P, A, and B of the valve are connected with positive pressure and inlet/outlet ports of the air conveyor are connected with negative pressure. Two thermal-type flow sensors were used to indicate the steady flow rate in the air circuit and vacuum circuit, respectively. A proportional directional control valve (abbreviated as “control valve” in the following) was connected in the vacuum circuit to adjust the suction flow rate. During the experiments, the supply pressure was regulated to 500 kPa, and a choked flow rate of 100 L/min was produced using an orifice. In this case, the pressure in the buffer tank was approximately 10 kPa. For the vacuum branch, the supply pressure was −20 kPa. A software package was developed on the computer using VC++ (Microsoft, Redmond, WA, USA) to receive and process the images transmitted from the camera. Electrical control signals were sent by computer via multichannel digital output boards with a 5 V/24 V amplifier circuit.

### 5.2. Control Method

**Figure 12:**
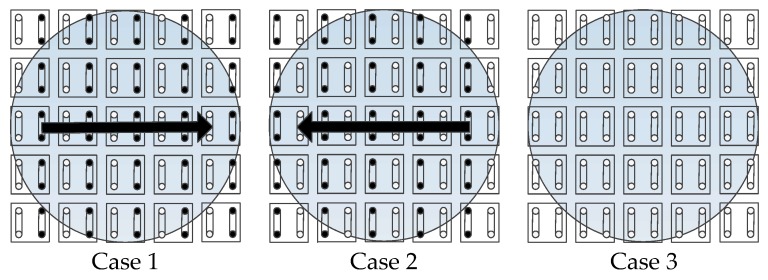
Three cases for the actuating direction.

**Figure 13:**
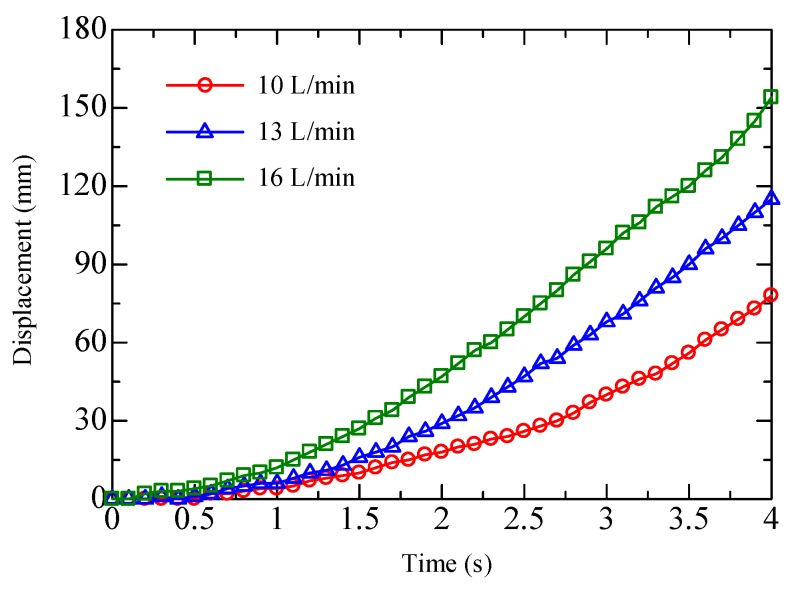
Displacement versus time during open loop control experiment.

In this work, the direction and the amplitude of the actuating force are separately controlled. The actuating direction can be selected by opening and closing the appropriate valves. Figure 12 shows three different cases. The small filled black circle represents that the port is selected for negative pressure while the empty circle for positive pressure. For cases 1 and 2, the arrow denotes the direction of the generated force. For case 3, all of the ports are supplied with positive pressure and therefore there is no actuating force generated. Within the covered area, the ports are selected for the control pressure by switching the fast switching valves. However, for the uncovered area, all of the ports are connected with positive pressure but no pressurized air film can be formed. The aforementioned analysis on the viscous force implies that the amplitude of the actuating force can be adjusted by varying the suction flow rate. To verify this, open loop control experiments were performed to compare the movements of the object with different suction flow rates. The test object is a circular plate (diameter: φ = 80, weight: 16.9 g), which covers five columns of actuating cells. The supply flow rate is set to 100 L/min, and the suction flow rate is set to 10, 13, and 16 L/min, respectively. Figure 13 shows the measured results in the form of displacement versus time. Evidently, the object exhibits a larger velocity as the suction flow rate increases, and the velocity at 4 s is approximately 37.5 mm/s, 51.6 mm/s, and 80.1 mm/s. As a result, we can accelerate, decelerate, and reverse the movement by adjusting the actuating force and the direction.

**Figure 14:**
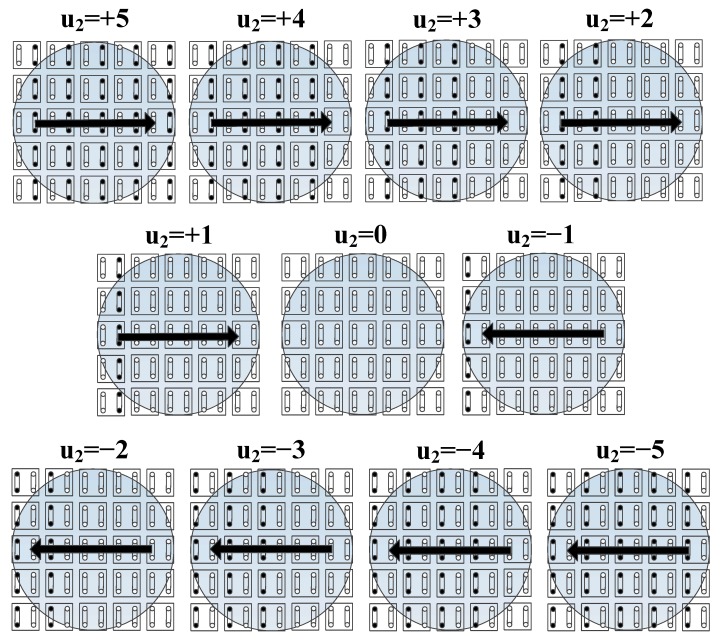
Definition of the control signal for method 2.

**Figure 15:**
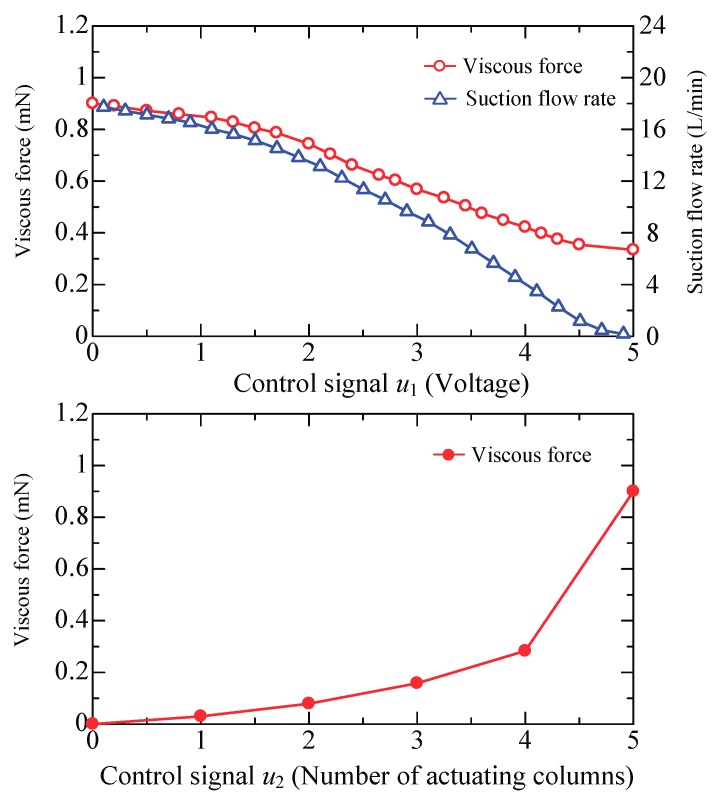
Relationship between the viscous force and the control signal.

The actual control signal *u* sent to the control valve is an analog voltage that ranges from 0 to 5 V. The upper plot in Figure 15 shows the relations for *F*-*u*_1_ and *Q*_2_-*u*_1_. The suction flow rate, *Q*_2_, changes from 18 to 0 L/min, and the viscous force approximately changes from 0.9 mN to 0.3 mN. We observe that the viscous force cannot be adjusted to zero by only changing the suction flow rate while the inlet flow rate is kept unchanged. This means that a continuous control signal can be sent when the object is far away from the target position, but it has to be changed to the discrete mode when the object is adequately close to the desired position. The authors’ previous work [32,33,34] implies that another method using the number of active actuating columns (e.g., an integer from 0 to +5) as the control signal also makes sense. Figure 14 shows examples of the control signal (an integer) from +5 to −5. The small filled black circle represents a port connected to negative pressure and the empty circle represents a port connected to positive pressure. Naturally, the actuating force is enlarged as the number of active actuating columns increases. The object can slow down or reverse motion by switching the positive pressure and negative pressure for the appropriate ports. The lower plot in Figure 15 shows the relation of *F*-*u*_2_. The discrete-valued output control is effective if the sampling period is sufficiently small.

Two control methods are introduced above: (1) selecting the number of actuating columns by fast switching valves (abbreviated as “method 2” in the following). In this method, both the actuating direction and amplitude of the viscous force can be controlled only with the fast switching valves; (2) adding an extra control valve to adjust the suction flow rate (abbreviated as “method 1” in the following). In this method, a servo valve is connected to the vacuum branch to adjust the suction flow rate thereby changing the amplitude of the viscous force while the actuating direction is controlled using the fast switching valves. An example is raised here for a more clear explanation for the control signals. For method 1, *u*_1_ = 1 means that an analog voltage of 1 V is sent to the control valve. For method 2, *u*_2_ = 1 means that only the leftmost column of cells is used as the active actuating column (refer to Figure 14). 

**Figure 16:**
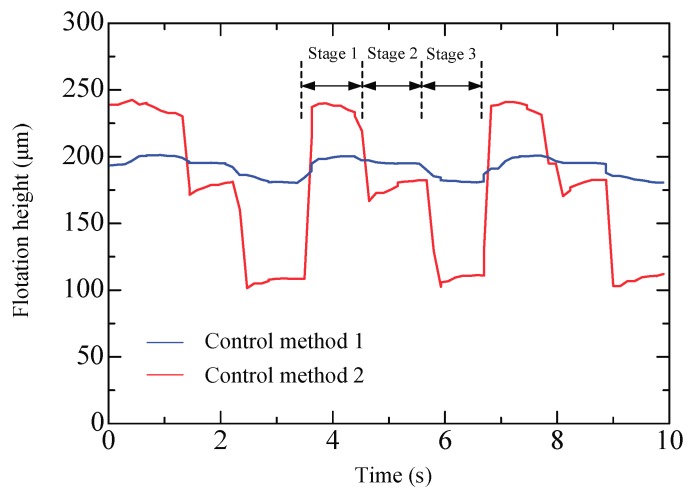
Fluctuations of the floating object by the two control methods.

We thus conduct experiments to compare the two control methods. A circular plate (diameter: φ = 80, weight: 10.4 g) is floated on the air conveyor, and a smooth wall is set to prevent it from slipping away. The control signals are sent and the fluctuations of the floating object are recorded. For method 2, the control signals (5, 4, and 3) are sent by turns and held for a while (5, 4, and 3 for stages 1, 2, and 3, respectively), while for method 1, an analog voltage corresponding to the actuating force, which are roughly the same as that of the method 2, are sent. Figure 16 shows the experimental results. When the control signal is switched, the flotation height for method 2 exhibits an exaggerative fluctuation over 100 μm, which is induced by the sudden flow. But for method 1 it is only 20 μm. In the case that an object is being transported, the fast switching valves need to be frequently switched in order to vary the viscous force. Because the moving object does not contact the device, it is very likely to slip way when subjected to large disturbances. From this perspective, we believe that method 1 is comparatively superior and it is employed in the current work. 

The PID controller is used for 1D position control. The position of the object on the conveyor surface, which is required to settle on a reference value, is measured continuously by the camera. The error between the measured signal and the reference signal is used as feedback. The control structure is given in Equations (17) and (18):

(17) $$e(t)=y_{\mathrm{ref}}-y\;$$

(18) $$u(t)=K_{p}e(t)+K_{i}T\int_{0}^{t}e(\tau )d\tau +\frac{K_{d}}{T}\dot{e}(t)\;$$

where *e* is the error between the reference position (*y*_ref_) and the measured position (*y*), *K*_p_ is the proportional gain, *K*_i_ is the integral gain, *K*_d_ is the derivative gain, and *T* is the sampling time.

The image is processed at a rate of 50 frames per second so that the sampling time is 0.02 s. The coefficients *K*_p_, *K*_i_, and *K*_d_ are obtained by trial-and-error in order to achieve a good performance. The control signal *u* is calculated by the controller and its value is limited to a range of −5–5. The sign indicates the actuating direction, which is changed using the fast switching valves, and the amplitude related to the actuating force, are adjusted by the control valve.

### 5.3. Results and Discussion

**Figure 17:**
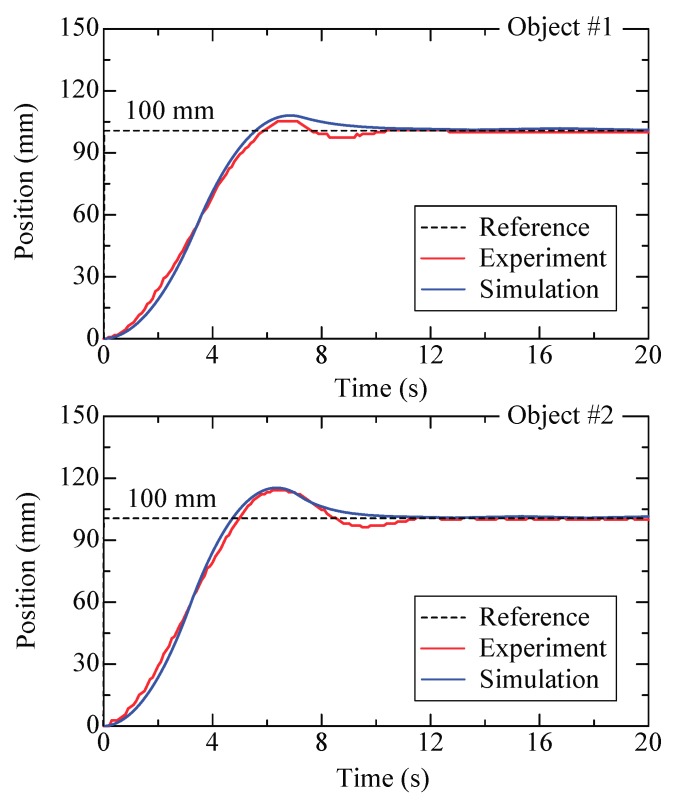
Simulated and experimental results for step response.

Experimental control has been made with two planar objects and the designed PID controller. The two objects have a diameter of 80 mm and a weight of 16.9 g and 10.4 g, designated as #1 and #2, respectively. During the experiments, the initial rest position of the object is set at 0 and the desired position is set as 100 mm. Results are shown in Figure 17 and the performances are summarized in Table 1. The rise time is almost the same for the two objects (3.7 s and 3.6 s). The overshoot for the heavy object exhibits a bigger value (13.3%) than that of the light object (7.3%). The static error is commonly over 0.3 mm due to the limited resolution of the used camera, and if an advanced camera is in use it can be improved. Simulated results are provided in comparison with the experimental results. In the simulation, dynamic characteristics of the valve are neglected because its response is much faster than that of the system. Furthermore, the relation of the floating height against the suction flow rate for the test object was experimentally measured and has been included. However, flow perturbation, which occurs at the opening and closing of the valves, has not been taken into account. This, to a certain extent, causes a difference between the simulations and the experimental results. Nevertheless, observation shows that the simulated curve is in accordance with the experimental data, and this verifies the effectiveness of the theoretical modeling and control method. The PID parameters slightly change with different objects because the controller is not adequately robust. Note that the use of steps as reference signals allows for the understanding of performances in the most unfavorable case. Taking a smooth reference signal would obtain a better result. It should be noted that there are some limitations for the size of the transported object. In theory, transport of large glass substrates with areas of approximately 2–3 m^2^ is feasible if the designed air conveyor is adequately large. However, the current air conveyor has an effective area of 228 × 110 mm^2^ (16 × 8 square pockets distributed in arrays), and therefore, for a circular plate, the maximum diameter should be less than 110 mm. Meanwhile, the minimum size for the plate should ensure the coverage of four complete pockets for stable movements. Therefore, the minimum diameter should be larger than 34 mm. In addition, the weight of the object produces a noticeable effect on the floatation height and thereby affects the actuating force. In this work, for a φ = 50 mm plate, transport is difficult to realize if the object’s weight is less than 5 g.

**Figure 18:**
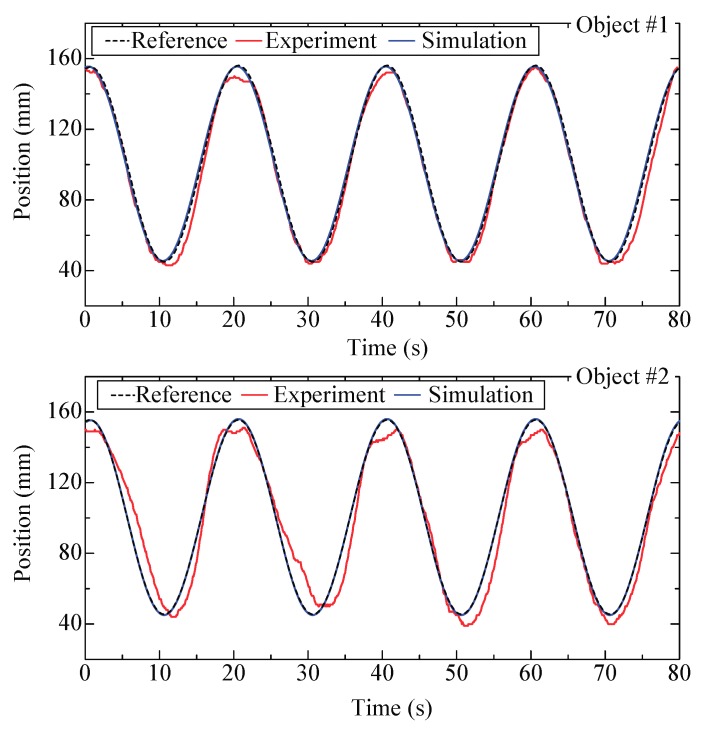
Simulated and experimental results for position tracking.

Figure 18 shows the tracking results. The reference signal is a sine signal with a period of 20 s and amplitude of 55 mm. The results show that good tracking is realized but large deviations unexpectedly appear. This is because the object is transported without any mechanical contact and even tiny disturbance might exert influences on the tracking results. Theoretically, a tracking frequency of 0.1 Hz is viable; however, it is difficult to experimentally actualize due to unavoidable disturbances. These results are compared with those of a previous work [33,34]. For the step response, the overshoot is negligibly small in the previous work but considerably large in the current work. This is due to differences in the experimental apparatus. In the previous work, a smooth wall that touches the edge of the object is set in order to make the object move along a straight line, and thus friction exists. The maximum velocity was 5.2 mm/s and the maximum acceleration was 1.6 mm/s^2^. However, in the current work, the geometry of the air conveyor and the control method have been optimized and such a block is not required. Overshoot easily occurs in the absence of friction, but the maximum velocity was increased to 17.2 mm/s, while the maximum acceleration increased to 5.4 mm/s^2^. In addition, the relative error evaluated by the root-sum-square method was approximately11.5% for the previous work and 3.2% for the current work. From the comparisons, we conclude that the method with suction flow rate as the control variable, rather than the number of actuating columns, is superior because it can reduce the disturbance induced by the opening and closing of the valves, and thus the object moves more smoothly. Better results are expected if disturbances can be further reduced, and this will be a part of future works.

## 6. Conclusions

In this study, a completely contactless air conveyor system is developed to transport and position planar objects. A simplified model derived from the film flow behavior is proposed to correlate the viscous force with the suction flow rate and the gap thickness. Experimental setups were established to measure the film pressure distribution and the viscous force, and the basic characteristics were studied. The film pressure is symmetrically distributed in the width direction but nonsymmetrically distributed in the length direction. This means that only the viscous force along the airflow direction in the pockets needs consideration. Moreover, the total viscous force is the composition of an actuating force generated in the pocket areas and the side areas and a drag force generated in the dam area. The viscous force increases if the suction flow rate is enlarged or the gap thickness is narrowed. Actually, the viscous force exhibits a more significant variation than expected as did the change of the suction flow rate, which would indirectly influence the floating height of the object and thus reinforce the effects. Comparison of the experimental results and the simulated results indicates that the model can accurately predict the viscous force, which is necessary for the motion simulation. A PID controller was applied for 1D-position control and position tracking. The direction and the amplitude of the actuating force are separately controlled. The control method that varies the suction flow rate rather than the number of the actuating columns was used because it can greatly reduce the disturbance to the floating object.

In comparison with the previous works by the research group of TU Delft [27,28,29,30] and R. Zeggari et al. [17], the highlights of the present work mainly lie in the aspects of control method, improvements to modeling, and some measuring methods as follows.

(1) The actuating direction and the amplitude of the viscous force are separately controlled, with switching valves used for selecting the actuating direction and a servo valve for varying the suction flow rate. This control method can effectively reduce the flow perturbation which easily occurs at the opening and closing of the fast-switching valves.
(2) An extension to the modeling is made using a fitting equation to approximate the effective flow rate through the gap for the case that the suction flow rate is changeable.
(3) Apparatuses for measuring the film pressure distribution and conducting position control of planar objects were developed. The model-based calculated results agree with the experimental results indicating that the measuring method is feasible.

## Figures and Tables

**Table 1:** Step response performance of the PID controller.

Object	#1	#2
Mass (g)	10.4	16.9
Step amplitude (mm)	100	100
*K*_p_	0.08	0.07
*K*_i_	0.005	0.005
*K*_d_	1.5	1.2
Rise time (s)	3.7	3.6
Overshoot	7.3%	13.3%
Static error (mm)	0.45	0.35
